# Dendritic Cells and T Cells Interact Within Murine Afferent Lymphatic Capillaries

**DOI:** 10.3389/fimmu.2019.00520

**Published:** 2019-03-22

**Authors:** Morgan Campbell Hunter, Alvaro Teijeira, Riccardo Montecchi, Erica Russo, Peter Runge, Friedemann Kiefer, Cornelia Halin

**Affiliations:** ^1^Institute of Pharmaceutical Sciences, ETH Zürich, Zurich, Switzerland; ^2^Max Planck Institute for Molecular Biomedicine, Münster, Germany; ^3^European Institute for Molecular Imaging – EIMI, Westfälische Wilhelms-Universität Münster, Münster, Germany

**Keywords:** dendritic cells, T cells, immune interactions, lymphatic vessels, adaptive immunity, migration

## Abstract

Afferent lymphatic vessels contribute to immunity by transporting antigen and leukocytes to draining lymph nodes (LNs) and are emerging as new players in the regulation of peripheral tolerance. Performing intravital microscopy in inflamed murine ear skin we found that migrating dendritic cells (DCs) and antigen-experienced effector T cells spend considerable time arresting or clustering within afferent lymphatic capillaries. We also observed that intralymphatic T cells frequently interacted with DCs. When imaging polyclonal T cells during an ongoing contact-hypersensitivity response, most intralymphatic DC-T cell interactions were short-lived. Conversely, during a delayed-type-hypersensitivity response, cognate antigen-bearing DCs engaged in long-lived MHCII-(I-A/I-E)-dependent interactions with antigen-specific T cells. Long-lived intralymphatic DC-T cell interactions reduced the speed of DC crawling but did not delay overall DC migration to draining LNs. While further consequences of these intralymphatic interactions still need to be explored, our findings suggest that lymphatic capillaries represent a unique compartment in which adaptive immune interaction and modulation occur.

## Introduction

Afferent lymphatic vessels are present within most vascularized tissues and functionally convey lymph toward and into a draining LN. By transporting soluble inflammatory mediators, antigens and leukocytes, afferent lymphatic vessels establish an immunological connection between peripheral tissues and LNs. In addition to these traditional transport functions, several emerging studies highlight the role of lymphatic endothelium itself as a key modulator of peripheral immune responses (1–3).

The main cell types migrating via afferent lymphatic vessels are antigen-experienced CD4^+^ T cells and antigen-presenting dendritic cells (DCs) (3, 4). While T cell recirculation through afferent lymphatic vessels is thought to contribute to immunesurveillance, DC migration is important for maintenance of tolerance and for induction of protective immunity in draining LNs (3). In this regard, DCs within the tissue take up antigen and migrate via afferent lymphatic vessels to a draining LN. Within the LN, naïve T cells survey arriving DCs for presentation of antigen. In the case that a naïve T cell encounters a cognate antigen, the T cell undergoes clonal expansion and differentiation into effector and memory T cells. Intravital microscopy has revealed that such adaptive DC-T cell interactions progress through distinct phases of contact that depend on factors such as antigen recognition, timing of activation, signal strength, and the inflammatory environment (5, 6). At the end of the proliferation and differentiation phase, antigen-experienced effector, and memory T cells exit the LN via efferent lymphatic vessels and migrate to inflamed peripheral tissues. There, effector/memory T cells may be re-stimulated by antigen to perform local effector functions, or exit the tissue via afferent lymphatic vessels. Performing intravital microscopy in the murine ear skin we and others have recently described that both DCs and CD4^+^ effector/memory T cells spend several hours actively patrolling within initial capillaries and are only passively transported to the draining LN once they reach the larger downstream collecting vessels (7–10). Considering the long time spent in lymphatic capillaries and the emerging knowledge of the immune-modulatory functions of lymphatic vessels (1, 3), we here set out to further characterize the intralymphatic migratory behavior of DCs and T cells and to specifically investigate whether these cells might interact inside lymphatic capillaries.

## Materials and Methods

### Mouse Strains

Wilde-type (WT) C57BL/6 mice, VE-cadherin-Cre × RFP (7), hCD2-DsRed × Prox1-GFP (10), Prox1-Orange × CD11c-YFP (11, 12), and hCD2-DsRedxOTII (13, 14) mice were crossed and/or bred in specific-pathogen-free (SPF) facilities in-house. I-A/I-E^−/−^ mice (15) were acquired from an SPF facility at University of Zurich Laboratory Animal Services Center (LASC), Schlieren. All experiments were approved by the Cantonal Veterinary Office Zurich.

### Bone Marrow Chimeras

Bone marrow chimeras were generated as described in (7).

### Generation of BM-DCs

WT or I-A/I-E^−/−^ BM-DCs were generated as described in Supplemental Experimental Procedures.

### Intravital Microscopy Specifications

Intravital microscopy of mouse ear pinna was performed as previously described (10). Exact imaging conditions and cell motility specifications are listed in Supplemental Experimental Procedures. For adoptive transfer experiments: five to six hours prior to imaging, mice were anesthetized using isoflurane (2–5%) and 500'000 to 750'000 WT or I-A/I-E^−/−^ labeled BM-DCs, or CD11c-YFP BM-DCs, were adoptively transferred into the ear skin in 2-3 injections of up to 5 μl each.

### Analysis of DC and T Cell Contacts

For DC - T cell contact analysis, individual DCs were followed frame-by-frame and contact with T cells manually annotated. Direct contact for longer than 2 min was considered an active interaction. Contacts shorter than 2 min were excluded from the analysis. A gap size of 2 min between contacts with the same T cell was considered a single continuing contact. Consequently, gaps in contact for more than 2 min were considered independent contacts. Using these criteria, a contact plot for each DC was generated. The length of each contact, DC occupancy and number of T cell contacts per DC were analyzed. DC occupancy index = a measure of the percentage time that a DC is contacted by a T cell/s during an imaging period.

### Flow Cytometry

Flow cytometry was performed on ear skin, LNs or BM-DCs as described in Supplemental Experimental Procedures.

### CHS-Induced or DTH-Induced Inflammation

See Supplemental Experimental Procedures.

### Statistical Analysis

All cell tracking data are presented as medians and all other results presented as mean plus standard deviation. Data sets were analyzed using Prism 7 (GraphPad). Kruskal-Wallis test followed by post hoc analysis was used for multiple comparisons and Mann-Whitney *U*-test for simple comparisons.

## Results

### DCs Patrol and Arrest Within Lymphatic Capillaries

**Figure 1:**
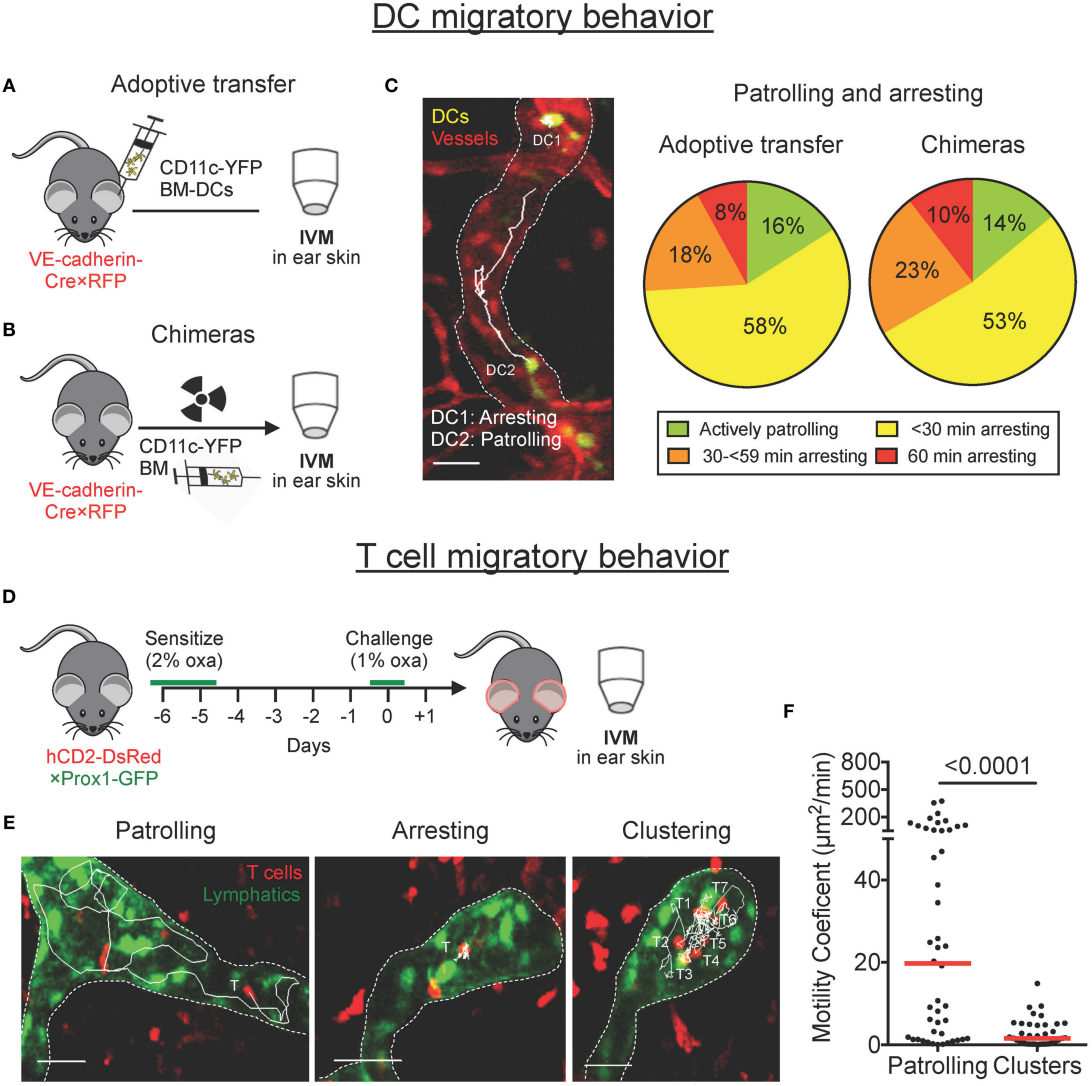
DCs patrol and arrest, and T cells patrol, cluster and arrest in lymphatic capillaries in mouse ear skin. **(A–C)** Intravital microscopy was performed in the ear skin of VE-cadherin-Cre × RFP mice in which YFP^+^ DCs had been adoptively transferred or in bone marrow chimeras. **(A,B)** Schematic diagrams of the experimental setups. **(C)** Representative image of YFP^+^ DC probing and patrolling migratory behavior (scale bars: 30 μm). Tracks of individual DCs imaged over 60 min are shown as solid white lines. Stopping times of CD11c-YFP DCs were quantified manually and classified into four groups based upon their migratory behavior within an imaging period of 60 min. Pooled data from 6–9 mice. **(D–F)** Intravital microscopy was performed in CHS-inflamed ear skin of hCD2-DsRed × Prox1-GFP mice. **(D)** Schematic diagram of the experimental setup. **(E)** Representative images of DsRed^+^ T cells migratory behavior inside lymphatic capillaries (scale bars: 30 μm). Tracks of individual T cells imaged over 30–45 min are shown as solid white lines. **(F)** Motility coefficient of patrolling T cells and T cells within clusters. Each dot represents a tracked cell. Median is shown as a red bar. Pooled data from 3 mice are shown.

We previously reported that DCs actively entered lymphatic capillaries and migrated in a semi-directed manner toward the collecting vessels (7, 9). Intriguingly, in these studies we also frequently observed DCs that remained arrested for long time periods. To assess the relative proportion of time that DCs spend arrested or patrolling, we classified DCs into four different groups, depending on whether they had spent the entire imaging period of 60 min (a) actively patrolling, (b) mainly patrolling, (c) mainly arrested, or (d) completely arrested. Imaging was performed in the ear skin of VE-cadherin-Cre × RFP mice, which feature red-fluorescent blood and lymphatic vessels (7), either upon adoptive transfer of LPS-matured YFP^+^ bone marrow-derived DCs (BM-DCs; Figure 1A), or upon reconstitution of these mice with bone marrow from CD11c-YFP mice (BM chimeras, Figure 1B). In chimeric animals, endogenous YFP^+^ DCs were induced to migrate into lymphatic vessels by intradermal LPS injection and topical imiquimod treatment. In both imaging setups only a small proportion of DCs (≈ 15%) continuously migrated or “patrolled” intravascularly, whereas a large proportion of DCs (≈ 75%) exhibited an intermittent arresting and patrolling behavior, and ~10% remained completely arrested throughout the imaging period (Figure 1C and Movie S1). Most notably, in both setups, around 30% of DCs were arrested for more than half of the imaging period (Figure 1C, orange and red sectors combined).

### T Cells Patrol, Cluster, and Frequently Arrest Within Lymphatic Capillaries

Performing intravital microscopy in contact hypersensitivity (CHS)-inflamed ear skin of hCD2-DsRed × Prox1-GFP mice, which feature red T cells and green lymphatic vessels (Figure 1D), we recently reported that, similarly to DCs, CD4^+^ effector/memory T cells entered into and actively patrolled within lymphatic capillaries in mouse ear skin (10). After further imaging and closer inspection of our videos, we also observed that several T cells remained arrested or clustered inside lymphatic capillaries (Figure 1E, Movie S2). Single-cell tracking analysis revealed that T cells in clusters were notably less motile than patrolling T cells (Figure 1F). Since clustering and swarming of T cells are hallmark phases of T cell activation in the draining LN (5, 6), we speculated that clustering or arresting T cells might be interacting with as yet “invisible” arrested DCs. In support of this “interaction hypothesis”, we also occasionally observed T cells interacting with motile GFP^+^ cells inside lymphatic capillaries of hCD2-DsRed × Prox1-GFP mice (Figures S1A,B, Movie S3). In flow cytometry a small fraction of GFP^+^ cells were found to be CD45^+^ cells and to express the DC markers CD11c and MHCII (I-A/I-E), indicating that the interacting cells might be DCs that had phagocytosed dying Prox1-GFP^+^ lymphatic endothelial cells (Figures S1C,D).

### Adoptively Transferred DCs Interact With T Cells Inside Lymphatic Capillaries During a CHS Response

To more definitively show that DCs interact with T cells inside lymphatic capillaries, we adoptively transferred YFP^+^ LPS-matured BM-DCs (Figure S2A) into the ear skin of hCD2-DsRed × Prox1-GFP mice, which had been inflamed by induction of a CHS response to oxazolone. As previously reported (7, 9), YFP^+^ DCs avidly entered and actively migrated within lymphatic capillaries. Most notably, YFP^+^ DCs frequently interacted with T cells inside lymphatic capillaries (Movie S4).

### The Majority of Intralymphatic Interactions Between T Cells and Transferred DCs Are Short-Lived and I-A/I-E^−/−^-independent During a CHS Response

**Figure 2:**
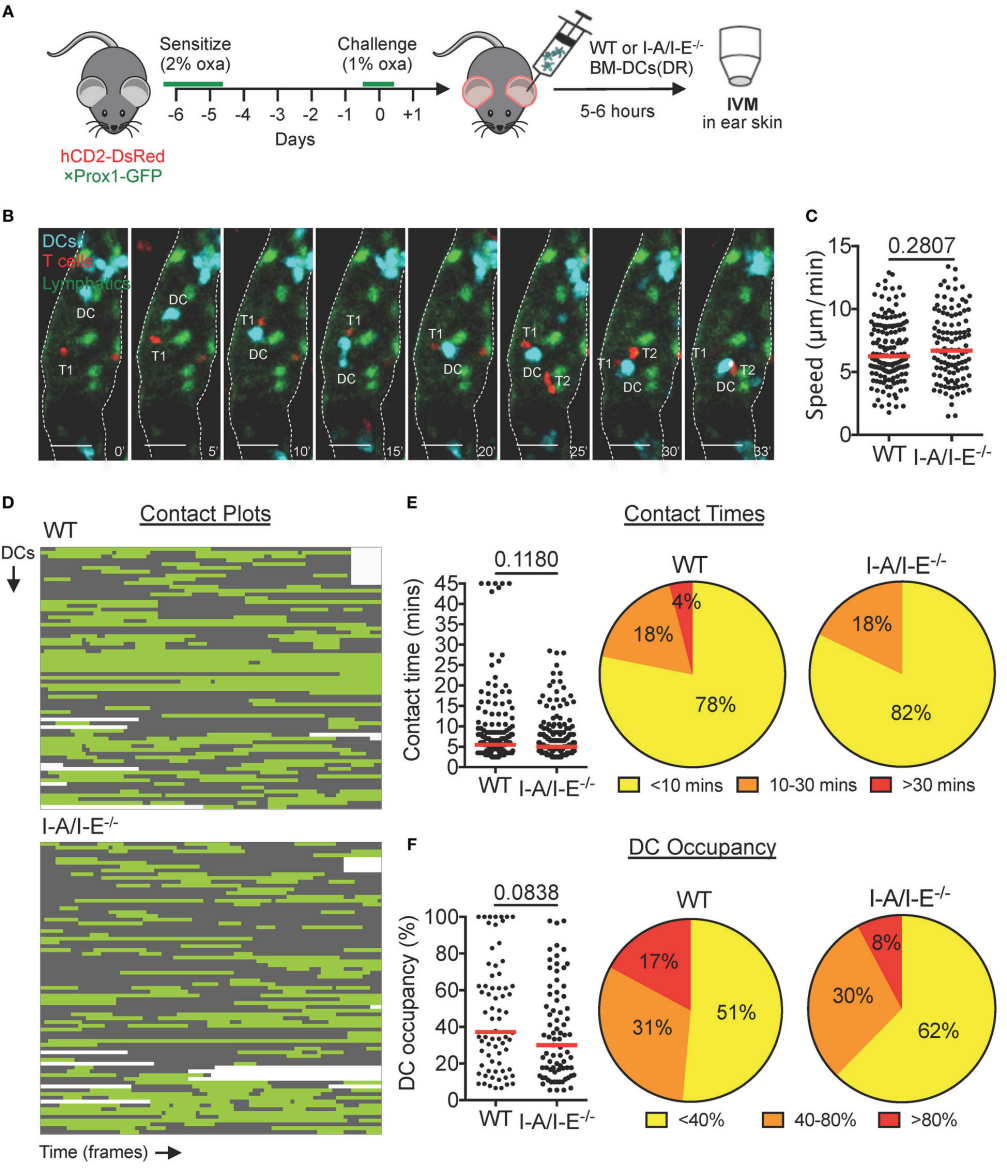
DCs interact with T cells inside lymphatic capillaries and short interactions are I-A/I-E-independent in CHS-inflamed mouse ear skin. **(A–F)** Intravital microscopy was performed in CHS-inflamed ear skin of hCD2-DsRed×Prox1-GFP mice after adoptive transfer of DeepRed-labeled WT or I-A/I-E^−/−^ BM-DCs. **(A)** Schematic diagram of the experimental setup. **(B)** Time-lapse images of a DeepRed^+^ WT DC (DC, cyan) contacting DsRed^+^ T cells (T1 and T2) inside a lymphatic capillary (scale bars: 30 μm). Times are shown in min. **(C)** Speed of WT and I-A/I-E^−/−^ DCs within lymphatic capillaries. **(D)** Plots of contact between WT and I-A/I-E^−/−^ DCs and T cells inside lymphatic capillaries. Each line is a DC indicating contact (green) and no contact (gray) with T cells. WT = 69 DCs, 174 contacts; I-A/I-E^−/−^ = 77 DCs, 196 contacts. **(E)** Quantitative analysis of contact times from **(C)** are shown individually and after classification into three contact time groups. Median is shown as a red bar. **(F)** The occupancy of DCs by T cells from **(C)** are shown individually and after classification into three groups. Each dot in **(C**,**E**,**F)** represents a tracked cell. Medians are shown as red bars. Pooled data from 6 mice per group are shown.

In LNs, migratory DCs are known to present processed antigen on MHCII (I-A/I-E) to circulating naïve T cells. To further characterize DC-T cell interactions inside dermal lymphatic capillaries, and to investigate their requirement for I-A/I-E, we adoptively transferred DeepRed-labeled wild-type (WT) or I-A/I-E^−/−^ BM-DCs into the CHS-inflamed ear skin of hCD2-DsRed × Prox1-GFP mice (Figures 2A,B). Activated WT and I-A/I-E^−/−^ DCs expressed similar levels of co-stimulatory molecules CD80 and CD86 (Figures S2B,C) and crawled with equal migratory speeds on lymphatic endothelial cell monolayers *in vitro* (Figure S2D) and in lymphatic capillaries of CHS-inflamed skin *in vivo* (Figure 2C). Both WT and I-A/I-E^−/−^ DCs interacted with T cells inside lymphatic capillaries, and in most cases intralymphatic DC-T cell interactions were dynamic in nature: DCs interacted with several T cells during the imaging period and frequently interacted with more than one T cell simultaneously (Figure 2B, Movie S5). To quantify intralymphatic DC-T cell interactions, we generated contact plots whereby interacting DCs were analyzed frame by frame for contact with T cells (Figure 2D). This assessment revealed that the majority (≈ 80%) of contacts were short-lived (<10 min), with only a handful (≈ 5%) of contacts lasting longer than 30 min (Figure 2E). No long-lasting contacts were observed for I-A/I-E^−/−^ DCs, but overall no major differences in T cell contact times between WT and I-A/I-E^−/−^ DCs were observed (Figure 2E). However, WT DCs showed a tendency to be more occupied by T cells than I-A/I-E^−/−^ DCs were (Figure 2F).

### Adoptively Transferred Antigen-Presenting DCs Engage in Prolonged Interactions With Cognate Intralymphatic T Cells During a Delayed-Type Hypersensitivity (DTH) Response

**Figure 3:**
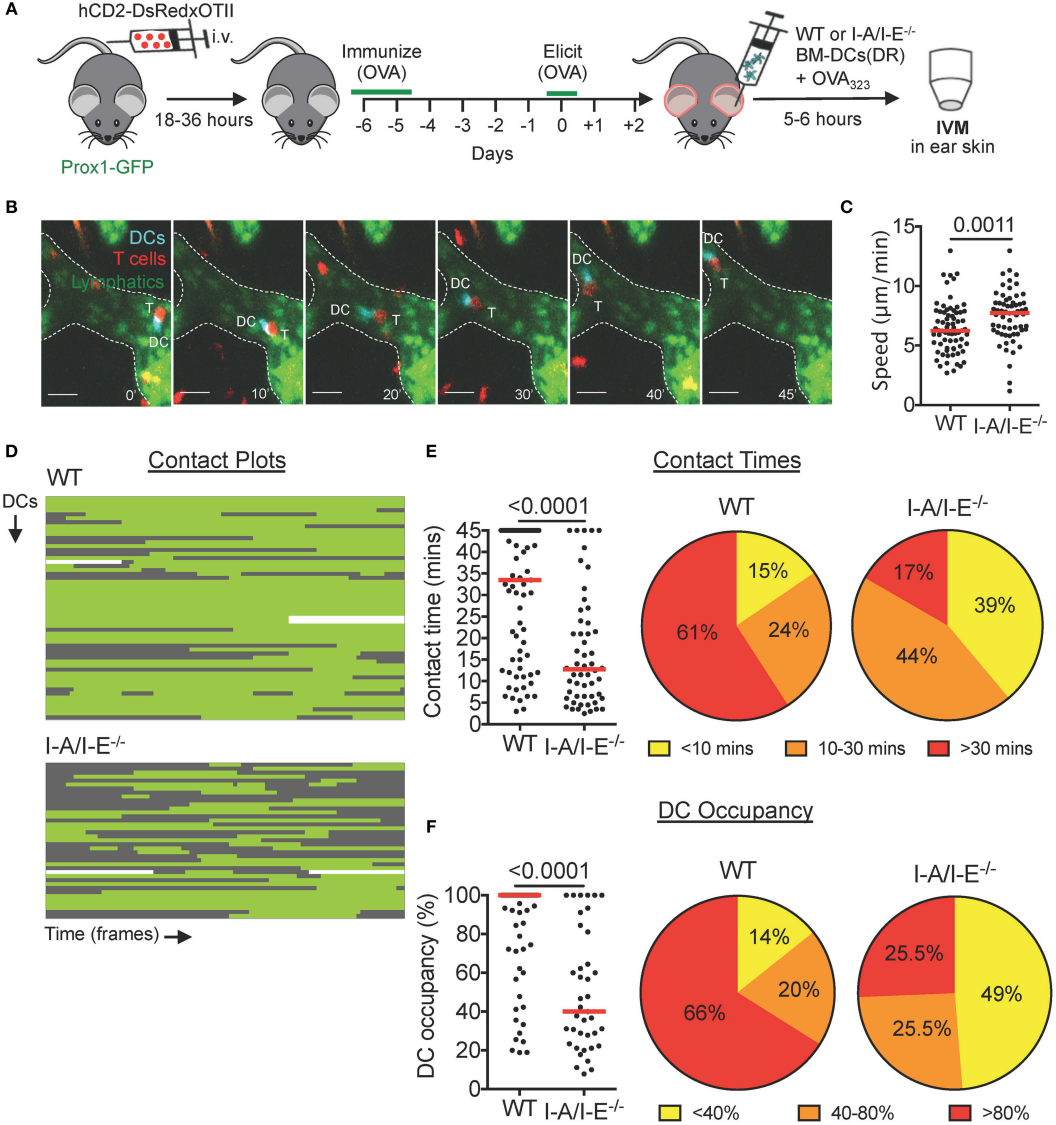
Prolonged intralymphatic DC-T cell interactions are I-A/I-E-dependent in DTH-inflamed mouse ear skin. **(A–F)** Intravital microscopy was performed in DTH-inflamed ear skin of Prox1-GFP mice in which DeepRed-labeled WT or I-A/I-E^−/−^ BM-DCs were adoptively transferred. **(A)** Schematic diagram of the experimental setup. **(B)** Time-lapse images of a DeepRed^+^ WT DC (DC, cyan) contacting a DsRed^+^ T cell (T) inside a lymphatic capillary (scale bars: 30 μm). Times are shown in min. **(C)** Speed of WT and I-A/I-E^−/−^ DCs within lymphatic capillaries. **(D)** Plots of contact between WT and I-A/I-E^−/−^ DCs and T cells inside lymphatic capillaries. Each line is a DC indicating contact (green) and no contact (gray) with T cells. WT = 56 DCs, 71 contacts; I-A/I-E^−/−^ = 39 DCs, 54 contacts. **(E)** Quantitative analysis of contact times from **(C)** are shown individually and after classification into three contact time groups. **(F)** The occupancy of DCs by T cells from **(C)** are shown individually and after classification into three groups. Each dot in **(C,E,F)** represents a tracked cell. Medians are shown as red bars. Pooled data from 3–4 mice/group each are shown.

Although not analyzed, most probably only a fraction of DsRed^+^ T cells recruited into the skin was hapten-specific in our CHS model (Figure 2). Moreover, considering that we had not exposed DCs to the CHS-inducing agent oxazolone prior to adoptive transfer, cognate DC-T cell interactions were unlikely to have been observed by intravital microscopy in this model. To overcome this limitation, we switched to investigating DC-T cell interactions during a DTH response in which only T cell receptor (TCR) transgenic, cognate antigen-specific T cells were DsRed^+^. To do so, we crossed TCR transgenic OTII mice, in which T cells are specific to ovalbumin-derived peptide OVA_323−339_ presented on I-A/I-E (14), with hCD2-DsRed mice. CD4^+^ T cells from hCD2-DsRed × OTII mice were transferred intravenously into Prox1-GFP mice, and mice were immunized with ovalbumin 1 day later (Figure 3A). After 4–7 days, ovalbumin was injected into the ears in order to elicit a DTH response (Figure 3A). Two days after elicitation, mouse ears were visibly reddened and ear thickness had increased (Figure S3A). By intravital microscopy we observed many DsRed^+^ T cells within the tissue and inside lymphatic capillaries (Figure S3B). Characterization of the T cell population in DTH-inflamed ears revealed that DsRed^+^OTII T cells constituted ≈ 5–20% of CD4^+^ T cells in the ear skin (Figures S3C,D,E).

With a working DTH model, we adoptively transferred DeepRed-labeled, OVA_323−339_-pulsed WT or I-A/I-E^−/−^ BM-DCs into the DTH-inflamed ear skin of our model mice (Figures 3A,B). In comparison to the CHS model (Figure 2), contacts between WT DCs and T cells were less dynamic in nature: although WT DCs occasionally contacted more than one T cell, the majority of WT DCs engaged in long-lived contacts with a single T cell (Figures 3D,E, Movie S6). Most notably, more than 60% of contacts between WT DCs and T cells lasted longer than 30 min (Figure 3E). Conversely, the majority of contacts between I-A/I-E^−/−^ DCs and T cells were short-lived, with <20% of contacts lasting longer than 30 min (Figure 3E). Moreover, whereas more than 65% of WT DCs were occupied by T cells for more than 80% of their track duration, only around 25% of I-A/I-E^−/−^ DCs were equally occupied by T cells (Figure 3F). Consequently, intralymphatic I-A/I-E^−/−^ DCs migrated faster than their WT counterparts (Figure 3C). However, in a competitive homing experiment, adoptively transferred WT and I-A/I-E^−/−^ DCs migrated equally well to the draining LN (Figure S4).

### Endogenous DCs Interact With T Cells Inside Lymphatic Capillaries During a DTH Response to Ovalbumin

**Figure 4:**
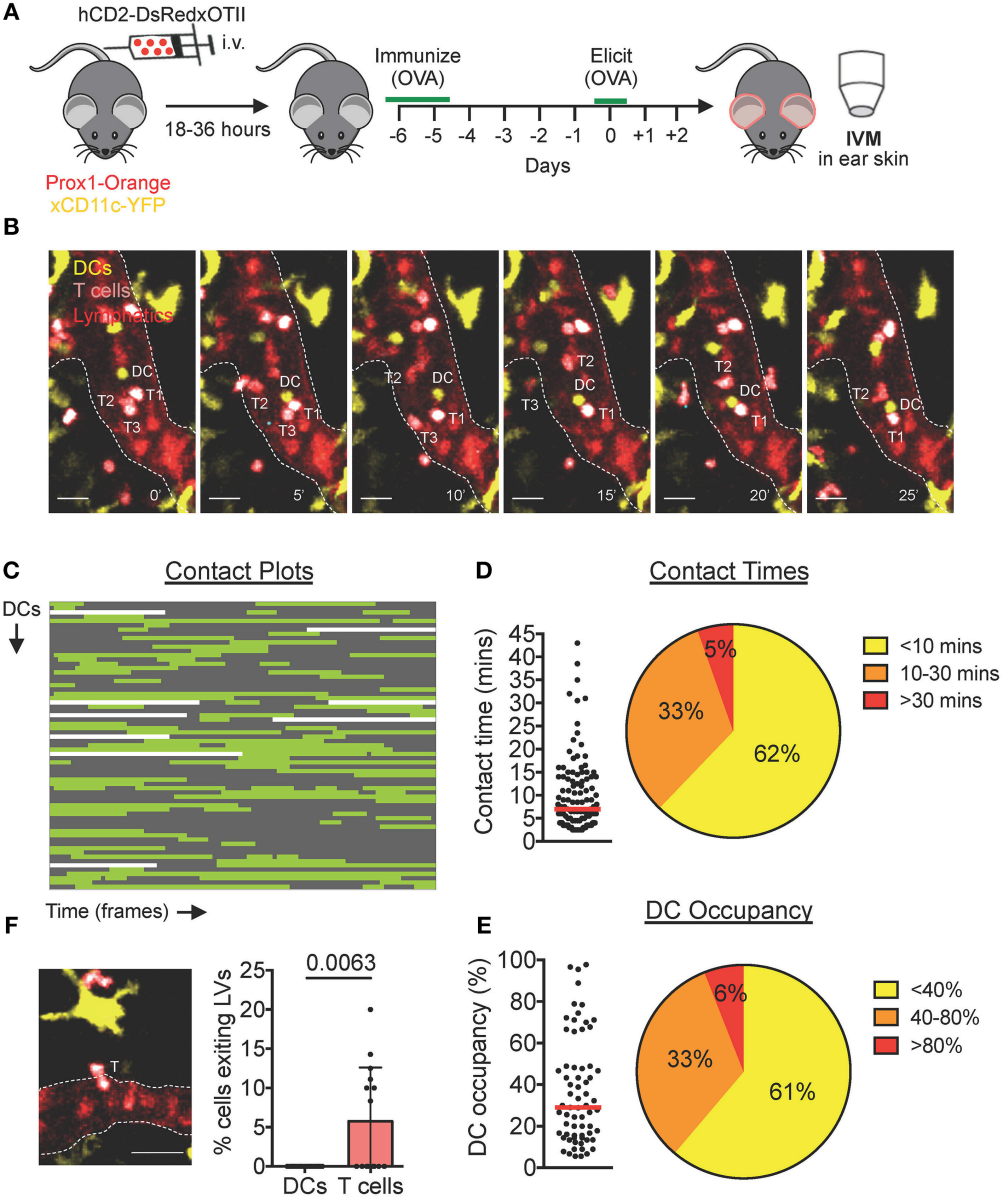
Endogenous DCs interact with T cells inside lymphatic capillaries in DTH-inflamed mouse ear skin. **(A–F)** Intravital microscopy was performed in DTH-inflamed ear skin of Prox1-Orange×CD11c-YFP mice. **(A)** Schematic diagram of the experimental setup. **(B)** Time-lapse images of a YFP^+^ DC (DC, yellow) contacting DsRed^+^ T cells (T1 and T2) inside a lymphatic capillary (scale bars: 30 μm). A third T cell (T3) is shown exiting a lymphatic capillary. Times are shown in min. **(C)** Plots of contact between DCs and T cells inside lymphatic capillaries. Each line is a DC indicating contact (green) and no contact (gray) with T cells. Sixty seven DCs, 111 contacts. **(D)** Quantitative analysis of contact times from **(C)** are shown individually and after classification into three contact time groups. **(E)** The occupancy of DCs by T cells from **(C)** are shown individually and after classification into three groups. Each dot in **(D)** and **(E)** represents a tracked cell. Medians are shown as red bars. **(F)** Intravital microscopy snapshot of a DsRed^+^ T cell (T) exiting a lymphatic capillary (scale bar: 30 μm) and quantification of the percentage of intralymphatic DCs or T cells that exited a lymphatic capillary during an imaging period. Each dot represents a movie analyzed. Mean and standard deviation are shown. Pooled data from 5 mice are shown.

To investigate whether also endogenous DCs interact with T cells inside lymphatic capillaries, we established our hCD2-DsRed × OTII DTH model in Prox1-Orange × CD11c-YFP mice, which feature orange lymphatic vessels and yellow DCs (Figure 4A). Two days after challenge with ovalbumin, both endogenous YFP^+^ DCs and *in vivo*-expanded CD4^+^ hCD2-DsRed × OTII cells could be observed actively migrating and interacting inside lymphatic capillaries (Figure 4B, Movie S7). Analysis of contact plot data (Figure 4C) revealed that the majority of DCs engaged in short-lived contacts (<10 min) with T cells, with only a small percentage (5%) of DCs engaging in contacts longer than 30 min (Figure 4D). Although several DCs contacted more than one T cell, the majority of DCs engaged in a single contact with a T cell during an imaging period (Figure 4C). Consequently, the majority of DCs were occupied by a T cell/s for <40% of their track duration (Figure 4E).

### T Cells, but Not DCs, Can Exit Lymphatic Capillaries in Murine Skin

Upon close inspection of videos generated from our CHS and DTH imaging setups, we occasionally observed T cells that exited lymphatic capillaries back into the surrounding tissue (Figure 4F, Movie S8). Egress across the endothelium was brief (≈ 2–5 min) and T cells visibly squeezed their cell body in order to exit the lymphatic lumen (Figure 4F, Movie S8). Although these events were not seen in every video, quantification in our endogenous DTH setup revealed that in total, ≈ 5–10% of intralymphatic T cells exited capillaries during an imaging period of 45 min (Figure 4F). Conversely, in all our imaging experience over the years, and quantitatively shown in our endogenous DTH setup (Figure 4F), we never observed a DC exit a lymphatic capillary.

## Discussion

In this study we have used intravital microscopy to further detail the behavior of DCs and T cells within dermal lymphatic capillaries during an ongoing immune response. In agreement with previous reports by us and by others (7–10) DCs and T cells actively migrated and patrolled within lymphatic capillaries. Moreover, we found that both cell types frequently arrested or clustered within lymphatic capillaries and that T cells, but not DCs, occasionally exited from the vessel lumen back into the tissue. Most intriguingly, we observed the occurrence of intralymphatic DC-T cell interactions.

Interactions between DCs and T cells are crucial for mounting an adaptive immune response. While they have mostly been studied in the draining LN during priming of naïve T cells (5, 6), only a few studies have investigated interactions of DCs with antigen-experienced effector/memory T cells in peripheral tissues like the skin (16–18). Given the spatial confinements of lymphatic vessels and the enhanced recruitment of activated DCs and T cells and drainage of antigen into the vessels during inflammatory processes, afferent lymphatic vessels might provide an ideal local compartment for adaptive modulation of the ongoing immune response. Intriguingly, several previous studies analyzing leukocyte subsets in afferent human lymph already reported the presence of cell aggregates comprising DCs and IFNγ-secreting CD4^+^ T cells (4, 19–21), indicating that also in humans DC-T cell interactions might be occurring in afferent lymphatic vessels.

When eliciting a DTH response toward ovalbumin in mice with DsRed^+^ TCR-transgenic OT-II T cells the majority of adoptively transferred OVA_323_-_339_ peptide-presenting DCs engaged in long-lived, MHCII (I-A/I-E)-dependent interactions with TCR transgenic OTII T cells (Figure 3). By contrast, when imaging polyclonal DsRed^+^ T cells 24 h after elicitation of a CHS response—a setup where likely only few of the adoptively transferred (unpulsed) DCs were presenting a cognate, haptenated antigen—numerous short-lived but only few long-lived DC-T cell interactions were observed (Figure 2). Similarly, imaging in our endogenous model of an ovalbumin-induced DTH response 48 h after ovalbumin injection (Figure 4), long-lived interactions only occurred in 5% of all cases. The reason why not more long-lived endogenous DC–T cell interactions occurred may be linked with the (unknown) level of OVA_323−339_ peptide presentation: In contrast to the BM-DC transfer experiments, where imaging was carried out shortly after transfer of OVA_323−339_ peptide pulsed DCs (Figure 3), likely much less OVA_323−339_ was present on endogenous intralymphatic DCs when imaging 2 days after ovalbumin challenge (Figure 4). Overall antigen availability has been recognized as an important determinant of the duration of DC-T cell contacts in other studies (22–24). Moreover, somewhat in line with our findings, interactions within the tissue of DTH-inflamed rat ear skin were shown to progress from long-lived contacts during onset to less frequent short-lived contacts during the peak of the response (17).

At this point we do not know the specific subset of T cells involved in the observed intralymphatic DC-T cell interactions, and we can only speculate about the potential immunological significance of these interactions. Given current knowledge of T cell trafficking through inflamed afferent lymphatic vessels (3, 25) it is likely that intralymphatic DC-T cell interactions either involve CD4^+^ effector T cells or regulatory T cells (T_regs_). During an ongoing immune response, both effector T cells and T_regs_ are recruited into peripheral tissues irrespective of their antigenic specificity (18, 26, 27). By contrast, exit from the inflamed tissues via lymphatic vessels appears to be at least in part dependent on whether or not the T cell encountered its cognate antigen while surveying the interstitial space (28, 29). Particularly in the initial phase of a developing immune response (e.g., an infection), when antigen is still scarcely distributed, cognate effector T cells might not encounter their antigen on antigen-presenting cells scanned in the tissue and hence exit into lymphatics. In the case that an effector T cell now encountered a cognate antigen-presenting DC within lymphatic capillaries, the effector T cell could be re-activated and instructed to exit the lymphatic vessel back into surrounding tissue and continue searching for antigen in order to exert its effector functions in the tissue. Considering this scenario, it is intriguing that we found a substantial fraction of intralymphatic T cells exiting the vessel again (Figure 4). Overall, this exiting behavior could contribute to immunosurveillance, as these cells would take a “short-cut” back into tissue where their cognate antigen might be located, rather than recirculating through draining LNs, lymphatic vessels, and blood. In any case, our finding of T cells exiting back into the tissue asks for a revision of the current lymphatic trafficking paradigm, in which afferent lymphatic vessels have thus far exclusively been regarded as cellular tissue exit routes.

Interestingly, besides effector T cells, T_regs_ were found to constitute ~50% of T cells emigrating from CHS-inflamed skin to draining LNs via afferent lymphatic vessels (30, 31). Moreover, T_regs_ arriving via afferent lymphatic vessels were shown to contribute to the suppression of immunity in draining LNs and to be important for preventing exacerbated CHS-induced inflammation in skin (30, 31). In addition to directly suppressing T cell priming (32), T_regs_ are capable of suppressing the maturation phenotype of antigen-presenting DCs (33). Considering that afferent capillaries accumulate both DCs and T_regs_ that are exiting from the tissue, it is perceivable that DC-T_reg_ interactions that modulate the DC phenotype might already take place in this compartment. Given the availability of T_reg_-specific reporter mice, such as FoxP3-GFP mice (34), this hypothesis could be investigated in the near future.

We also observed that endogenous intralymphatic DCs or T cells frequently clustered amongst themselves. While we occasionally found several clustering T cells interacting with DCs, DC-T cell interactions do not seem to explain the formation of all or larger T cell clusters. Homotypic T cell-T cell clusters have previously been described within LNs and have been shown to augment T cell activation and differentiation via paracrine signaling of IL-2 and IFN-γ (35, 36). Although we cannot yet specifically determine the activation status of clustering T cells within lymphatic vessels, this might become possible in the future using new photoconvertible systems, such as Kaede mice (37).

Our simultaneous imaging of DCs and T cells also revealed that not all immotile DCs or T cells were necessarily engaging in interactions with other DCs or T cells, but that some cells simply remained arrested on the lymphatic endothelium for long time periods. It is tempting to speculate that DCs and T cells might be exchanging immune-modulatory signals with lymphatic endothelium during these lengthy arrest and interaction periods. Interestingly, autoantigen-presenting lymphatic endothelial cells in LNs have been identified as important players in the regulation of peripheral CD8^+^ T cell tolerance (1, 38). Moreover, LN lymphatic endothelial cells have been shown to archive exogenous antigen derived from viral infections or vaccinations and pass it on to migratory DCs capable of antigen cross-presentation (39). Besides impacting CD8^+^ T cell responses, emerging studies have also identified a role for LN lymphatic endothelial cells in modulating CD4^+^ T cell responses, either by directly presenting antigen to T cells or by transferring self-antigen to DCs, leading to the induction of T cell anergy and tolerance (40, 41).

Of interest, we have observed that similarly to LN lymphatic endothelial cells, lymphatic endothelial cells in afferent lymphatic vessels upregulate PDL-1 and MHCII in response to inflammation [(42) and data not shown]. Together with our intravital microscopy observations of lengthy DC and T cell arresting, this could suggest that lymphatic endothelial cells in afferent lymphatics might exert similar immune-modulatory functions as in draining LNs. In fact, in the case of DCs, the production of prostaglandins (43) and ICAM-1 by lymphatic endothelial cells of afferent lymphatic vessels (44) were already suggested to impact maturation of migratory DCs. Moreover, dermal lymphatic vessels have recently been identified as important players in the regulation of peripheral CD8^+^ T cell tolerance during tumor growth (2). Given the extensive time that DCs and T cells spend inside afferent lymphatics during their exit from the tissue, future studies should investigate the expanding role of afferent lymphatic endothelium in the immunomodulation of intralymphatic passengers.

In summary, our study for the first time reports the occurrence of adaptive DC-T cell interactions within lymphatic capillaries. Combined with the current literature, our findings provide several further pieces of evidence suggesting that afferent lymphatic vessels represent more than just a trafficking route to draining LNs but rather a new compartment for adaptive immune interactions and immune modulation.

## Data Availability

All datasets generated for this study are included in the manuscript and/or the Supplementary Files.

## Author Contributions

MH designed research, performed research, analyzed data, and wrote the paper. AT designed research, performed research and analyzed, and discussed data. RM, ER, and PR performed research and analyzed and discussed data. FK provided mice and discussed data. CH designed research, analyzed data, and wrote the paper.

### Conflict of Interest Statement

The authors declare that the research was conducted in the absence of any commercial or financial relationships that could be construed as a potential conflict of interest.

## References

[1] Humbert M Hugues S Dubrot J. Shaping of peripheral T cell responses by lymphatic endothelial cells. Front Immunol. (2016) 7:684. 10.3389/fimmu.2016.00684 28127298 PMC5226940

[2] Lane RS Femel J Breazeale AP Loo CP Thibault G Kaempf A. IFNγ-activated dermal lymphatic vessels inhibit cytotoxic T cells in melanoma and inflamed skin. J Exp Med. (2018) 215:3057–74 10.1084/jem.20180654 30381467 PMC6279400

[3] Permanyer M Bosnjak B Forster R. Dendritic cells, T cells and lymphatics: dialogues in migration and beyond. Curr Opin Immunol. (2018) 53:173–9. 10.1016/j.coi.2018.05.004 29857205

[4] Yawalkar N Hunger RE Pichler WJ Braathen LR Brand CU Human afferent lymph from normal skin contains an increased number of mainly memory / effector CD4(+) T cells expressing activation, adhesion and co-stimulatory molecules. Eur J Immunol. (2000) 30:491–7. 10.1002/1521-4141(200002)30:2<491::AID-IMMU491>3.0.CO;2-H 10671204

[5] Miller MJ Safrina O Parker I Cahalan MD. Imaging the single cell dynamics of CD4+ T cell activation by dendritic cells in lymph nodes. J Exp Med. (2004) 200:847–856. 10.1084/jem.20041236 15466619 PMC2213293

[6] Mempel TR Henrickson SE Von Andrian UH. T-cell priming by dendritic cells in lymph nodes occurs in three distinct phases. Nature. (2004) 427:154–9. 10.1038/nature02238 14712275

[7] Nitschke M Aebischer D Abadier M Haener S Lucic M Vigl B. Differential requirement for ROCK in dendritic cell migration within lymphatic capillaries in steady-state and inflammation. Blood. (2012) 120:2249–58. 10.1182/blood-2012-03-417923 22855606

[8] Tal O Lim HY Gurevich I Milo I Shipony Z Ng LG. DC mobilization from the skin requires docking to immobilized CCL21 on lymphatic endothelium and intralymphatic crawling. J Exp Med. (2011) 208:2141–53. 10.1084/jem.20102392 21930767 PMC3182054

[9] Russo E Teijeira A Vaahtomeri K Willrodt AH Bloch JS Nitschke M. Intralymphatic CCL21 promotes tissue egress of dendritic cells through afferent lymphatic vessels. Cell Rep. (2016) 14:1723–34. 10.1016/j.celrep.2016.01.048 26876174

[10] Teijeira A Hunter MC Russo E Proulx ST Frei T Debes GF. T cell migration from inflamed skin to draining lymph nodes requires intralymphatic crawling supported by ICAM-1/LFA-1 interactions. Cell Rep. (2017) 18:857–65. 10.1016/j.celrep.2016.12.078 28122237

[11] Hagerling R Pollmann C Kremer L Andresen V Kiefer F. Intravital two-photon microscopy of lymphatic vessel development and function using a transgenic Prox1 promoter-directed mOrange2 reporter mouse. Biochem Soc Trans. (2011) 39:1674–81. 10.1042/BST20110722 22103506

[12] Lindquist RL Shakhar G Dudziak D Wardemann H Eisenreich T Dustin ML. Visualizing dendritic cell networks *in vivo*. Nat Immunol. (2004) 5:1243–50. 10.1038/ni1139 15543150

[13] Veiga-Fernandes H Coles MC Foster KE Patel A Williams A Natarajan D. Tyrosine kinase receptor RET is a key regulator of Peyer's patch organogenesis. Nature. (2007) 446:547–51. 10.1038/nature05597 17322904

[14] Barnden MJ Allison J Heath WR Carbone FR. Defective TCR expression in transgenic mice constructed using cDNA-based alpha- and beta-chain genes under the control of heterologous regulatory elements. Immunol Cell Biol. (1998) 76:34–40. 10.1046/j.1440-1711.1998.00709.x 9553774

[15] Kontgen F Suss G Stewart C Steinmetz M Bluethmann H. Targeted disruption of the MHC class II Aa gene in C57BL/6 mice. Int Immunol. (1993) 5:957–64. 10.1093/intimm/5.8.957 8398989

[16] Natsuaki Y Egawa G Nakamizo S Ono S Hanakawa S Okada T. Perivascular leukocyte clusters are essential for efficient activation of effector T cells in the skin. Nat Immunol. (2014) 15:1064–9. 10.1038/ni.2992 25240383

[17] Matheu MP Beeton C Garcia A Chi V Rangaraju S Safrina O. Imaging of effector memory T cells during a delayed-type hypersensitivity reaction and suppression by Kv1.3 channel block. Immunity. (2008) 29:602–14. 10.1016/j.immuni.2008.07.015 18835197 PMC2732399

[18] Egawa G Honda T Tanizaki H Doi H Miyachi Y Kabashima K. *In vivo* imaging of T-cell motility in the elicitation phase of contact hypersensitivity using two-photon microscopy. J Invest Dermatol. (2011) 131:977–9. 10.1038/jid.2010.386 21248770

[19] Brand CU Hunziker T Schaffner T Limat A Gerber HA Braathen LR. Activated immunocompetent cells in human skin lymph derived from irritant contact dermatitis: an immunomorphological study. Br J Dermatol. (1995) 132:39–45. 10.1111/j.1365-2133.1995.tb08622.x 7756150

[20] Olszewski WL Grzelak I Ziolkowska A Engeset A. Immune cell traffic from blood through the normal human skin to lymphatics. Clin Dermatol. (1995) 13:473–83. 10.1016/0738-081X(95)00087-V 8665458

[21] Brand CU Hunger RE Yawalkar N Gerber HA Schaffner T Braathen LR. Characterization of human skin-derived CD1a-positive lymph cells. Arch Dermatol Res. (1999) 291:65–72. 10.1007/s004030050385 10195392

[22] Henrickson SE Mempel TR Mazo IB Liu B Artyomov MN Zheng H. T cell sensing of antigen dose governs interactive behavior with dendritic cells and sets a threshold for T cell activation. Nat Immunol. (2008) 9:282–91. 10.1038/ni1559 18204450 PMC2698867

[23] Henrickson SE Perro M Loughhead SM Senman B Stutte S Quigley M. Antigen availability determines CD8(+) T cell-dendritic cell interaction kinetics and memory fate decisions. Immunity. (2013) 39:496–507. 10.1016/j.immuni.2013.08.034 24054328 PMC3914670

[24] Egen JG Rothfuchs AG Feng CG Horwitz MA Sher A Germain RN. Intravital imaging reveals limited antigen presentation and T cell effector function in mycobacterial granulomas. Immunity. (2011) 34:807–19. 10.1016/j.immuni.2011.03.022 21596592 PMC3164316

[25] Hunter MC Teijeira A Halin C. T Cell Trafficking through Lymphatic Vessels. Front Immunol. (2016) 7:613. 10.3389/fimmu.2016.00613 28066423 PMC5174098

[26] Stephens R Randolph DA Huang G Holtzman MJ Chaplin DD. Antigen-nonspecific recruitment of Th2 cells to the lung as a mechanism for viral infection-induced allergic asthma. J Immunol. (2002) 169:5458–67. 10.4049/jimmunol.169.10.5458 12421921

[27] Ghani S Feuerer M Doebis C Lauer U Loddenkemper C Huehn J. T cells as pioneers: antigen-specific T cells condition inflamed sites for high-rate antigen-non-specific effector cell recruitment. Immunology. (2009) 128:e870–e880. 10.1111/j.1365-2567.2009.03096.x 19740348 PMC2753954

[28] Gomez D Diehl MC Crosby EJ Weinkopff T Debes GF. Effector T cell egress via afferent lymph modulates local tissue inflammation. J Immunol. (2015) 195:3531–6. 10.4049/jimmunol.1500626 26355150 PMC4571282

[29] Mcnamee EN Masterson JC Veny M Collins CB Jedlicka P Byrne FR. Chemokine receptor CCR7 regulates the intestinal TH1/TH17/Treg balance during Crohn's-like murine ileitis. J Leukoc Biol. (2015) 97:1011–22. 10.1189/jlb.3HI0614-303R 25637591 PMC4438744

[30] Tomura M Honda T Tanizaki H Otsuka A Egawa G Tokura Y. Activated regulatory T cells are the major T cell type emigrating from the skin during a cutaneous immune response in mice. J Clin Invest. (2010) 120:883–93. 10.1172/JCI40926 20179354 PMC2827959

[31] Ikebuchi R Teraguchi S Vandenbon A Honda T Shand FH Nakanishi Y. A rare subset of skin-tropic regulatory T cells expressing Il10/Gzmb inhibits the cutaneous immune response. Sci Rep. (2016) 6:35002. 10.1038/srep35002 27756896 PMC5069467

[32] Schmidt A Oberle N Krammer PH. Molecular mechanisms of treg-mediated T cell suppression. Front Immunol. (2012) 3:51. 10.3389/fimmu.2012.00051 22566933 PMC3341960

[33] Dhainaut M Moser M. Mechanisms of surveillance of dendritic cells by regulatory T lymphocytes. Prog Mol Biol Transl Sci. (2015) 136:131–54. 10.1016/bs.pmbts.2015.08.003 26615095

[34] Bettelli E Carrier Y Gao W Korn T Strom TB Oukka M. Reciprocal developmental pathways for the generation of pathogenic effector TH17 and regulatory T cells. Nature. (2006) 441:235–8. 10.1038/nature04753 16648838

[35] Sabatos CA Doh J Chakravarti S Friedman RS Pandurangi PG Tooley AJ. A synaptic basis for paracrine interleukin-2 signaling during homotypic T cell interaction. Immunity. (2008) 29:238–48. 10.1016/j.immuni.2008.05.017 18674934 PMC4466225

[36] Gerard A Khan O Beemiller P Oswald E Hu J Matloubian M. Secondary T cell-T cell synaptic interactions drive the differentiation of protective CD8+ T cells. Nat Immunol. (2013) 14:356–63. 10.1038/ni.2547 23475183 PMC3962671

[37] Tomura M Yoshida N Tanaka J Karasawa S Miwa Y Miyawaki A. Monitoring cellular movement *in vivo* with photoconvertible fluorescence protein “Kaede” transgenic mice. Proc Natl Acad Sci USA. (2008) 105:10871–6. 10.1073/pnas.0802278105 18663225 PMC2504797

[38] Rouhani SJ Eccles JD Tewalt EF Engelhard VH. Regulation of T-cell tolerance by lymphatic endothelial cells. J Clin Cell Immunol. (2014) 5:1000242. 10.4172/2155-9899.1000242 25580369 PMC4286360

[39] Kedl RM Lindsay RS Finlon JM Lucas ED Friedman RS Tamburini BAJ. Migratory dendritic cells acquire and present lymphatic endothelial cell-archived antigens during lymph node contraction. Nat Commun. (2017) 8:2034. 10.1038/s41467-017-02247-z 29229919 PMC5725486

[40] Dubrot J Duraes FV Potin L Capotosti F Brighouse D Suter T. Lymph node stromal cells acquire peptide-MHCII complexes from dendritic cells and induce antigen-specific CD4(+) T cell tolerance. J Exp Med. (2014) 211:1153–66. 10.1084/jem.20132000 24842370 PMC4042642

[41] Rouhani SJ Eccles JD Riccardi P Peske JD Tewalt EF Cohen JN. Roles of lymphatic endothelial cells expressing peripheral tissue antigens in CD4 T-cell tolerance induction. Nat Commun. (2015) 6:6771. 10.1038/ncomms7771 25857745 PMC4403767

[42] Vigl B Aebischer D Nitschke M Iolyeva M Rothlin T Antsiferova O. Tissue Inflammation Modulates Gene Expression Of Lymphatic Endothelial cells and dendritic cell migration in a stimulus-dependent manner. Blood. (2011) 118:205–15. 10.1182/blood-2010-12-326447 21596851

[43] Christiansen AJ Dieterich LC Ohs I Bachmann SB Bianchi R Proulx ST. Lymphatic endothelial cells attenuate inflammation via suppression of dendritic cell maturation. Oncotarget. (2016) 7:39421–35. 10.18632/oncotarget.9820 27270646 PMC5129942

[44] Podgrabinska S Kamalu O Mayer L Shimaoka M Snoeck H Randolph GJ. Inflamed lymphatic endothelium suppresses dendritic cell maturation and function via Mac-1/ICAM-1-dependent mechanism. J Immunol. (2009) 183:1767–79. 10.4049/jimmunol.0802167 19587009 PMC4410990

