# Supplementary material for: Dendritic Cells and T Cells Interact Within Murine Afferent Lymphatic Capillaries
Source: Front Immunol. 2019 Mar 22;10:520. doi: 10.3389/fimmu.2019.00520 (PMC6440485; doi:10.3389/fimmu.2019.00520)
Supplement: Supplementary file 9 [file Data_Sheet_1.pdf]

SUPPLEMENTAL INFORMATION

Dendritic cells and T cells interact within murine afferent lymphatic capillaries

Morgan Campbell Hunter, Alvaro Teijeira, Riccardo Montecchi, Erica Russo, Peter Runge, Friedemann Kiefer, Cornelia Halin.

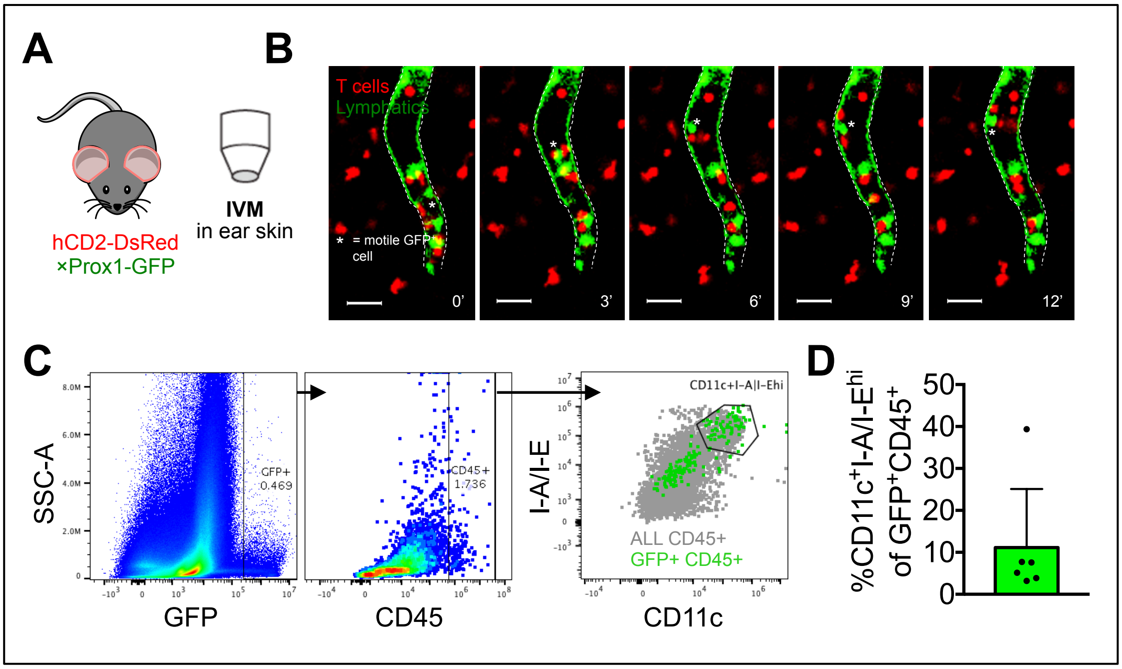

Figure S1: GFP$^+$ "phagocytes" interact with T cells inside lymphatic capillaries in CHS-inflamed mouse ear skin of hCD2-DsRed×Prox1-GFP mice. Related to Figure 1. (**A-B**) IVM was performed in CHS-inflamed ear skin of hCD2-DsRed×Prox1-GFP mice. (**A**) Schematic diagram of the experimental setup (same as Figure 1D). (**B**) Time-lapse images of a motile GFP$^+$ cell (*) contacting DsRed$^+$ T cells inside a lymphatic capillary (scale bars: 30 μm). Times are shown in minutes. (**C-D**) Flow cytometry analysis was performed on single-cell suspensions of CHS-inflamed ear skin of hCD2-DsRed×Prox1-GFP mice or Prox1-GFP mice. (**C**) Representative FACS plots showing the gating strategy for CD11c$^+$I-A/I-E^{hi} cells within the CD45$^+$ population of GFP$^+$ cells. All CD45$^+$ cells are shown as a control in grey. (**D**) Quantification of the data shown in (C). Mean and standard deviation are shown. Pooled data from 6 mice are shown.

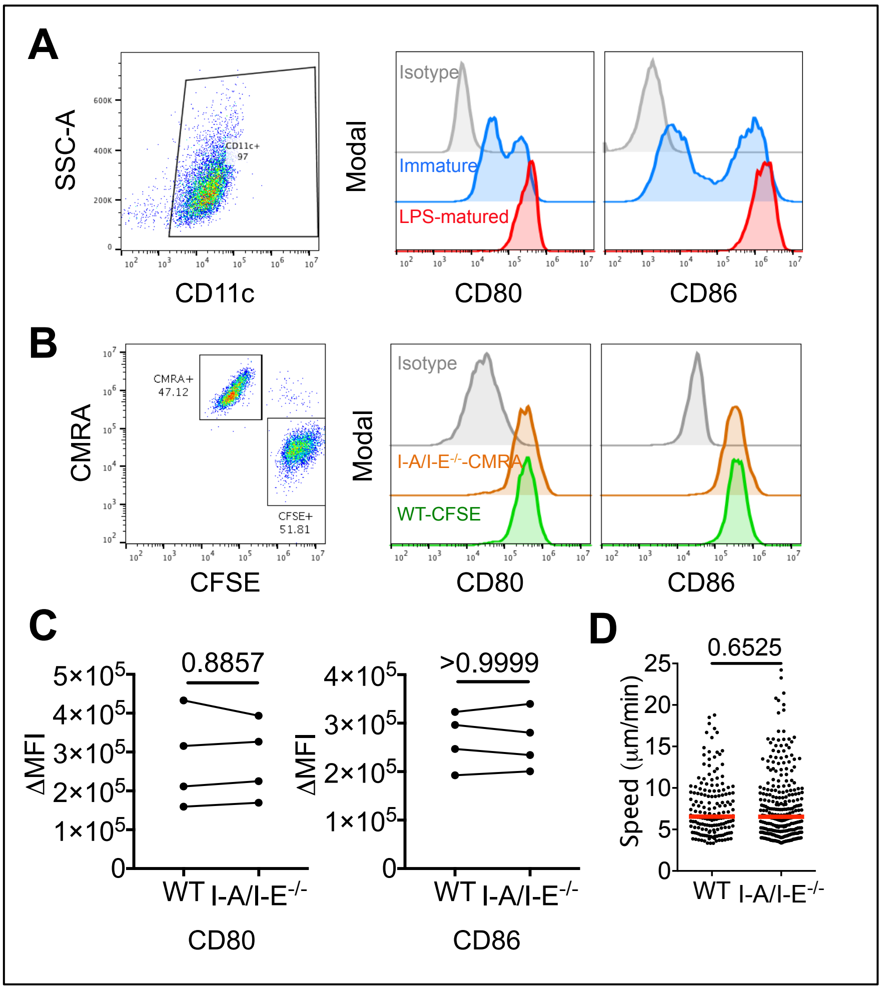

Figure S2: LPS-matured WT and I-A/I-E$^{-/-}$ BM-DCs express equal levels of co-stimulatory molecules CD80 and CD86 and migrate with an equal speed on lymphatic endothelium *in vitro*. Related to Figure 2. (**A**) Gating strategy and representative histograms of expression of CD80 and CD86 for immature (blue) and LPS-matured (red) WT BM-DCs. (**B-C**) CFSE-labeled WT and CMRA-labeled I-A/I-E$^{-/-}$ BM-DCs were mixed in a 1:1 ratio and analyzed for expression of co-stimulatory molecules CD80 and CD86. (**B**) Gating strategy and representative histograms of expression of CD80 and CD86 for I-A/I-E$^{-/-}$ (orange) and WT (green) BM-DCs. Isotype staining is shown in grey. Dyes were switched between experiments. Quantification from 4 independent experiments are shown in (**C**). (**D**) Speed of WT and I-A/I-E$^{-/-}$ BM-DCs *in vitro* (on imLEC monolayers). Each dot represents a tracked cell. Median is shown as a red bar. Pooled data from at least two similar experiments.

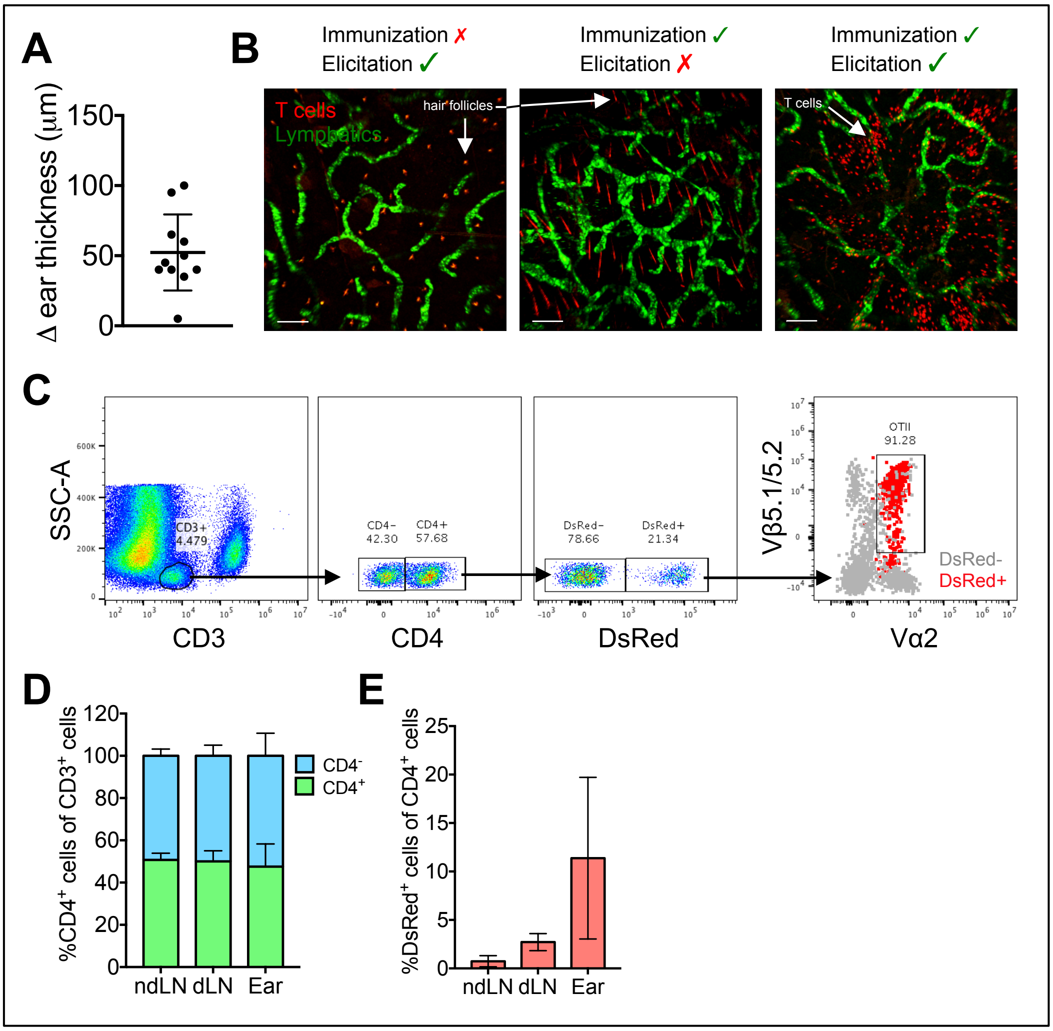

Figure S3: Characterization of a DTH response to ovalbumin in murine ear skin. Related to Figure 3. (**A**) Delta ear thickness, averaged per mouse, of DTH mice two days after elicitation. (**B**) 10X microscope images of mouse ear skin in DTH model mice in which either the immunization (left) or elicitation (middle) were omitted. In mice that were immunized, elicitation resulted in infiltration of hCD2DsRed×OTII T cells into the ear tissue (right). Scale bars, 200 µm. (**C**) Flow cytometry analysis was performed on single-cell suspensions of DTH-inflamed ear skin of model Prox1-GFP or C57BL/6 mice. Representative FACS plots showing the gating strategy for DsRed$^+$ cells within the CD4$^+$ population of CD3$^+$ cells. CD3^{hi} cells did not express CD4, Vα2, Vβ5.1/5.2 or DsRed and were suspected to be γδ T cells. Therefore these cells were excluded from the analysis. Right panel shows staining for Vα2 and Vβ5.1/5.2 on DsRed$^-$ (in grey) and DsRed$^+$ (in red) cells. (**D-E**) Quantification of (**D**) CD4$^+$ and CD4$^-$ cells within the CD3$^+$ population and (**E**) DsRed$^+$ cells within the CD4$^+$ population in the non-draining lymph node (ndLN), draining lymph node (dLN) and ear skin (Ear). Pooled data from 5-11 mice are shown.

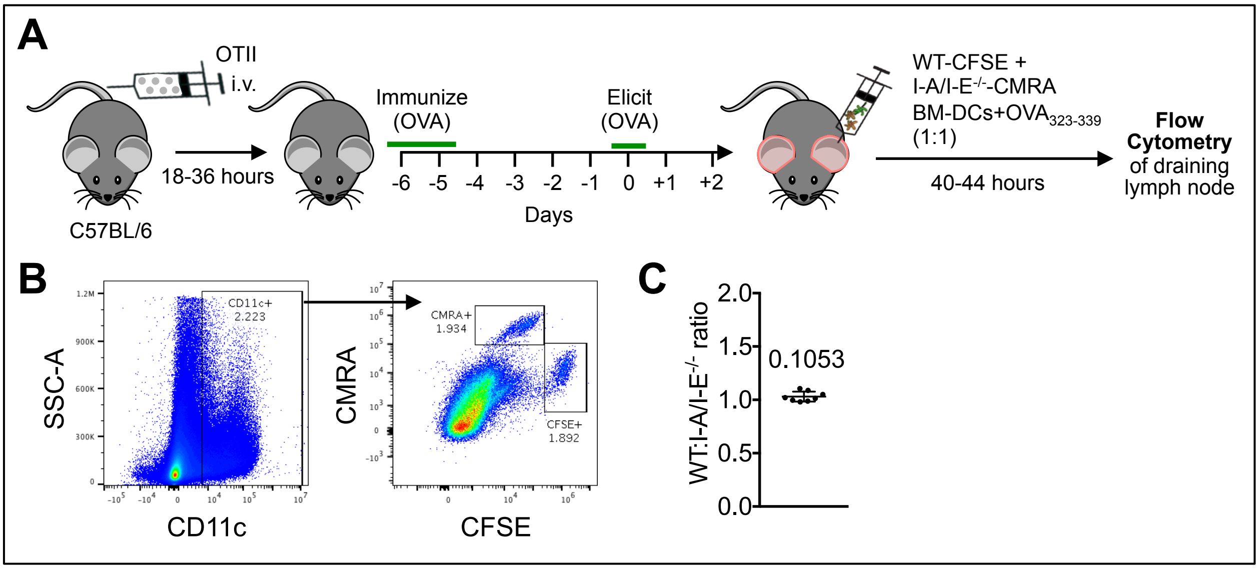

Figure S4: BM-DC migration from skin to dLN is I-A/I-E independent during a DTH response. Related to Figure 3. (**A-C**) An equal number of CFSE-labeled WT and CMRA-labeled I-A/I-E$^{-/-}$ BMDCs were adoptively transferred into the DTH-inflamed ear skin of C57BL/6 mice and 40-44 hours later the dLN harvested and the ratio of CFSE^{+} to CMRA^{+} BM-DCs assessed by flow cytometry. Fluorescent labels were switch between experiments. (**A**) Schematic of the experimental setup. (**B**) Representative flow cytometry plots of the % CFSE^{+} and CMRA^{+} cells of the CD11c^{+} population in the draining lymph node. (**C**) Ratio of WT: I-A/I-E$^{-/-}$ BM-DCs in the draining lymph node. Pooled data from 8 mice from 2 independent experiments. Ratio was statistically analyzed using a one sample t-test with a hypothetical value of 1.

SUPPLEMENTAL MOVIE LEGENDS

Movie S1: DCs patrol and arrest within lymphatic capillaries. Representative movie of DCs (yellow) patrolling and arresting behavior within lymphatic capillaries (red) in CHS-inflamed ear skin of bone marrow chimeric VE-cadherin-Cre×RFP mice reconstituted with bone marrow of CD11c-YFP mice. Tracks of selected DCs are shown in white. The movie was generated from 3D reconstructions of Z-Stacks. Movie specifications: 30 sec intervals, 10 frames/sec (= 300× accelerated).

Movie S2: T cells patrol, arrest and cluster within lymphatic capillaries. Representative movies of T cells (red) patrolling, arresting and clustering within lymphatic capillaries (green) in CHS-inflamed ear skin of hCD2-DsRed×Prox1-GFP mice. Tracks of selected T cells are shown in white. The movies were generated from 3D reconstructions of Z-Stacks. Movies specifications: 15 sec intervals, 20 frames/sec (= 300× accelerated).

Movie S3: GFP$^+$ "phagocytes" interact with T cells within lymphatic capillaries. Representative movies of GFP$^+$ "phagocytes" (green) interacting with T cells (red) within lymphatic capillaries (green) in CHS-inflamed ear skin of hCD2-DsRed×Prox1-GFP mice. The movies were generated from 3D reconstructions of Z-Stacks. Movies specifications: 15 sec intervals, 20 frames/sec (= 300× accelerated).

Movie S4: Adoptively transferred YFP$^+$ BM-DC interacting with endogenous T cells within lymphatic capillaries. Representative movie of an adoptively transferred CD11c-YFP$^+$ DC (yellow) interacting with endogenous T cells (red) within lymphatic capillaries (green) in CHS-inflamed ear skin of hCD2-DsRed×Prox1-GFP mice. The movie was generated from 3D reconstructions of Z-Stacks. Movie specifications: 30 sec intervals, 10 frames/sec (= 300× accelerated).

Movie S5: Short-lived interactions between adoptively transferred BM-DCs and endogenous T cells within lymphatic capillaries. Representative movies of adoptively transferred DeepRed-labeled DCs (cyan) engaging in short-lived interactions with endogenous T cells (red) within lymphatic capillaries (green) in CHS-inflamed ear skin of hCD2-DsRed×Prox1-GFP mice. The movies were

generated from 3D reconstructions of Z-Stacks. Movies specifications: 30 sec intervals, 10 frames/sec (= 300× accelerated).

Movie S6: Long-lived interactions between adoptively transferred BM-DCs and *in vivo* expanded OTII T cells within lymphatic capillaries. Representative movies of adoptively transferred $OVA_{323\text{-}339}$-pulsed DeepRed-labeled WT DCs (cyan) engaging in long-lived interactions with *in vivo* expanded hCD2-DsRed×OTII T cells (red) within lymphatic capillaries (green) in DTH-inflamed ear skin of Prox1-GFP mice. The movies were generated from 3D reconstructions of Z-Stacks. Movies specifications: 30 sec intervals, 10 frames/sec (= 300× accelerated).

Movie S7: Endogenous DCs interact with *in vivo* expanded OTII T cells within lymphatic capillaries. Representative movies of endogenous DCs (yellow) interacting with *in vivo* expanded hCD2-DsRed×OTII T cells (pink) within lymphatic capillaries (red) in DTH-inflamed ear skin of Prox1-Orange×CD11c-YFP mice. The movies were generated from 3D reconstructions of Z-Stacks. Movies specifications: 30 sec intervals, 10 frames/sec (= 300× accelerated).

Movie S8: T cells exiting lymphatic capillaries. Representative movies of hCD2-DsRed×OTII T cells (pink) exiting lymphatic capillaries (red) in DTH-inflamed ear skin of Prox1-Orange×CD11c-YFP mice. In the second movie, a T cell is seen exiting a lymphatic capillary shortly after contact with a DC (yellow). The movies were generated from 3D reconstructions of Z-Stacks. Movies specifications: 30 sec intervals, 10 frames/sec (= 300× accelerated).

SUPPLEMENTAL EXPERIMENTAL PROCEDURES

Bone marrow chimeras

Bone marrow chimeras were generated as described in (Nitschke et al., 2012). In brief, 4-6 week old VE-cadherin-Cre×RFP mice were irradiated (950 rad) and reconstituted with 5×10^6 bone marrow cells isolated from CD11c-YFP mice. Chimeric mice were used at least six weeks after irradiation. Endogenous DCs were induced to migrate into lymphatic vessels by intradermal LPS injection (one day prior to imaging) and topical imiquimod treatment (six hours prior imaging).

Generation of Bone Marrow-Derived DCs (BM-DCs)

BM-DCs were generated from CD11c-YFP, WT or I-A/I-E$^{-/-}$ mice by incubation of bone marrow cells in granulocyte-macrophage colony-stimulating factor (GM-CSF)-containing medium. In brief, 5×10^6 bone marrow cells, isolated from the tibia and femur of donor mice, were seeded into bacterial dishes (Greiner Bio-One, St. Gallen, Switzerland) in DC-medium containing RPMI 1640, 10% FBS, 15 nM HEPES, 1mM sodium pyruvate, penicillin (100 U/ml), streptomycin (100 µg/ml), L-glutamin (2 nM) (all purchased from GIBCO, Zug, Switzerland) 50 µg β-mercaptoethanol (Sigma) and GM-CSF derived from the supernatant of myeloma cells (X63 Ag8.653) transfected with murine GM-CSF cDNA (Zal et al., 1994). Every 2-3 days, cells were supplemented with new medium (5 ml medium was removed, centrifuged, and replaced with 10 ml fresh DC medium). On days 8-12 floating cells were transferred into tissue culture-treated dishes (TPP, Trasadingen, Switzerland) and incubated with 0.2 µg/ml lipopolysaccharide (LPS) (Enzo Life Sciences, Lausanne, Switzerland), and in some experiments, 100 nM $OVA_{323-339}$ (EMC microcollections, Tubingen, Germany). Floating cells were harvested the following day and DC purity and maturation was analyzed by flow cytometry for CD11c, I-A/I-E, CD80 and CD86. CD11c$^+$ purity = 95.27 ± 3.61 SD.

In vitro BM-DC flow chamber experiments

WT and I-A/I-E$^{-/-}$ BM-DCs matured with LPS (as outlined in "Generation of Bone Marrow-Derived DCs") were separately labeled with 10 µM CellTracker™ Green CMFDA (Invitrogen) or 10 µM CellTracker™ Orange CMRA (Invitrogen) in DC

medium (Gibco) for 20 minutes at 37 °C. Cells were washed with PBS and incubated in DC medium for 30 - 60 minutes at 37 °C to quench unreacted dye. BM-DCs were subsequently washed with PBS and centrifuged over an FBS gradient to remove dead cells. Immortalized murine lymphatic endothelial cells (imLECs) were cultured as outlined in (Teijeira et al., 2017). In brief, imLECs, expressing a heat-labile version of the large T antigen (Vigl et al., 2011), were cultured at 33°C on collagen (Advanced Biomatrix, San Diego, CA) and fibronectin (Millipore, Temecula, CA)-coated dishes (10 µg/ml each) in media containing 40% DMEM (low glucose), 40% F12-Ham, 20% FBS (all from Gibco), 56 µg/ml heparin (Sigma), 10 µg/ml endothelial cell mitogen (AbD Serotec, Duesseldorf, Germany), antibiotic antimycotic solution (1x; Fluka, Buchs, Switzerland), L-glutamin (2 nM; Fluka). Additionally, murine interferon-gamma (IFNγ; 1U/ml, Peprotech, London, UK) was added to induce large T-antigen expression (Vigl et al., 2011). Cultured imLECs were seeded into fibronectin and collagen (10 µg/ml each) coated channeled chamber slides (µ-Slide $VI^{0.4}$, IBIDI). Four hours later, chambers were connected to a peristaltic pump and flow media containing 40% DMEM (low glucose), 40% F12-Ham, 20% FBS, 10 mM HEPES (all from Gibco) and antibiotic antimycotic solution (1x; Fluka, Buchs, Switzerland) was applied with a shear stress of 0.015dyn/cm^2 for 16 hours to precondition imLECs. Flow was stopped, disconnected and 30’000 of a 1:1 mix of WT and I-A/I-$E^{-/-}$ BM-DCs were added on top of the channel. Immediately the channel was connected to flow and three minutes later flow was started at a rate of 0.015dyn/cm2. Images were acquired on a Nikon TE-2000E microscope (Tokyo, Japan) using DIC, FITC and RHOD fluorescent channels. Images were acquired every 30 second for 30 minutes and three videos were taken per experiment. Videos were analyzed with IMARIS software (v7.1.1, Bitplane, Zurich, Switzerland) and cells tracked using the IMARIS Spots and Tracks function. The following filters were applied: minimum displacement length = 15 µm; minimum track time = 10 minutes; minimum track length = 100 µm. Cells on the edge of the acquisition field, or cells migrating in groups were excluded from the quantification. Fluorescent labels were switched between experiments.

Intravital Microscopy Imaging

Intravital microscopy of the mouse ear pinna of VE-cadherin-Cre×RFP, hCD2-DsRed×Prox1-GFP and CD11c-YFP ×Prox1-mORANGE2 mice was performed as previously described (Nitschke et al., 2012, Russo et al., 2016, Teijeira et al., 2017).

Mice were anesthetized with an initial i.p. bolus injection of ketamine/medetomidine and ear hair removed by application of hair-removal cream for ≈ 2 mins. Anesthetized mice were transferred to a custom-made microscopy stage with the dorsal side of the ear position on the coverslip of the stage. A drop of PBS and second cover slip were placed on the ventral side of the ear and mice were placed on the imaging platform inside a 37 °C incubator. Further anesthesia was injected as required. Imaging was performed on a Leica SP8 confocal microscope (Carl Zeiss, Jena, Germany) equipped with a 20× 0.7 NA Plan Apochromat objective. An Argon laser (488 nm), solid-state 561 nm laser and solid-state 633 nm laser were used for GFP/YFP, RFP and DsRed and mORANGE2, and DeepRed excitation, respectively. Time-lapse images were acquired every 15 or 30 seconds at 512 × 512 pixels, no signal averaging. Image stacks typically ranged from 30 to 60 μm and featured 20 to 30 slices with individual slice depth typically between 1.5 to 2.5 μm. For discrimination between DsRed and mORANGE2 emissions in the same setup, collection filters were adjusted to optimally collect either a majority DsRed or mORANGE2 signal (DsRed = 570 to 630; mORANGE2 = 630 to 700). Overlapping of the two collection filters allowed sufficient discrimination between motile $DsRed^+$ T cells and static $mOrange2^+$ lymphatic vessels.

BM-DC Intravital Microscopy Imaging:

WT or $I\text{-}A/I\text{-}E^{-/-}$ BM-DCs matured with 200 ng/ml LPS, and in some experiments 100 nM $OVA_{323\text{-}339}$ (EMC Microcollections), overnight (as outlined in "Generation of Bone Marrow-Derived DCs") were labeled with 1 μM CellTracker™ Deep Red Dye (Invitrogen) in RPMI 1640 (Gibco) for 15 minutes at 37 °C. Cells were washed three times with PBS and incubated in DC medium for 30 - 40 minutes at 37 °C to quench unreacted dye. BM-DCs were subsequently washed with PBS and centrifuged over an FBS gradient to remove dead cells. Five to six hours prior to imaging, mice were anesthetized using isoflurane (2-5%) and 500'000 to 750'000 total WT or $I\text{-}A/I\text{-}E^{-/-}$ labeled BM-DCs, or CD11c-YFP BMDCs, were adoptively transferred into the ear skin in 2-3 injections of up to 5 μl each.

Analysis of DC and T cell motility

DC and T cell motility were analyzed as previously described (Russo et al., 2016, Teijeira et al., 2017). In brief, videos were analyzed with IMARIS software (v7.1.1,

Bitplane, Zurich, Switzerland) and cells were manually tracked using the IMARIS Spots and Tracks function. Cells with tracks <10 minutes were excluded from the analysis. Analysis criteria were calculated as follows: speed = total track length/track duration; motility coefficient = displacement^2/6*duration. Motility coefficient was defined as the propensity of a cell to move away from its point of origin. Migratory behaviors of DCs were quantified manually and separated into four groups.

Flow Cytometry

Flow cytometry was performed on ear skin, LNs, isolated CD4$^+$ cells or *in vitro* generated BM-DCs. Ear skin was digested with collagenase IV (Invitrogen) as previously described (Vigl et al., 2011). Single cell suspensions of skin or LNs were generated by passing tissue through a 40-µm cell strainer. Single-cell suspensions were stained using anti-mouse fluorophore-conjugated antibodies from Biolegend: CD45-AF700 or CD45-APC/Cy7 or CD45-PE/Cy7 (30-F11), CD3ε-APC (145-2C11), CD4-FITC or CD4-AF700 (GK1.5), TCR Vα2-Pacific Blue (B20.1), TCR Vβ5.1/5.2-PE/Cy7 (MR9-4), CD11b-PerCP (M1/70), CD11c-APC or CD11c-PE/Cy7 (N418), I-A/I-E-BV421 or I-A/I-E-PE (M5/114.15.2), CD80-FITC or CD80-PE or CD80-APC/Fire750 (16-10A1), CD86-PE or CD86-BV605 (GL-1). Respective isotype controls, unstained cells or FMO's were used for complex stainings. For ear tissue stainings, cells were pre-incubated with anti-CD16/CD32 (Biolegend) to reduce non-specific staining. When needed, dead cells were excluded from analysis by staining using Zombie Aqua™ Fixable Viability kit (Biolegend). Analysis was performed on a Beckman Coulter Cytoflex (4 lasers, 13 colors; Beckman Coulter) and analyzed offline using Flowjo software 10.4.2 (Treestar, Ashland, OR).

CHS-induced ear skin inflammation

A CHS response towards oxazolone was induced as previously described (Vigl et al., 2011). In brief, mice were anesthetized using isoflurane (2-5%) and sensitized by topical application of 2% oxazolone (4-ethoxymethylene-2-phenyl-2-oxazoline-5-one; Sigma) in acetone/olive oil (4:1 vol/vol) on the paws (5 µl) and shaved abdomen (50 µl). After 5 to 6 days, mice were anesthetized using isoflurane (2-5%) and challenged with 1% oxazolone solution topically applied on each side of the ears. Experiments were performed one day after challenge.

DTH-induced (ovalbumin) ear skin inflammation

LNs and spleens of OTII or hCD2DsRed×OTII mice were isolated and passed through a 40-µm-cell strainer. Red blood cells were lysed in ACK lysis buffer (150 mM NH_4Cl, 10 mM $KHCO_3$, 0.1 mM Na_2EDTA, pH 7.2-7.4) and washed in leukocyte medium (BM-DC medium without GM-CSF). $CD4^+$ cells were purified using CD4 (L3T4) microbeads (Miltenyi Biotec). Purified $CD4^+$ cells (>95% $CD4^+$ as assessed by flow cytometry) were resuspended in PBS and 0.8 – 1.2 million cells were injected intravenously into recipient mice. The following day, EndoFit™ Ovalbumin (InvivoGen) reconstituted in sterile endotoxin-free physiological water was emulsified at a 1:1 ratio with VacciGrade™ Complete Freund's Adjuvant (InvivoGen) and a total of 100 µg ovalbumin was injected in two points into the shaved belly skin of recipient mice. After 4 to 8 days, ovalbumin was mixed at a 1:1 ratio with PBS and a total of 50 µg ovalbumin was injected in three points into each ear of recipient mice. Characterization of the model and intravital microscopy experiments were performed two days after injection of ovalbumin (elicitation). Ear thickness of mice was measured using a caliper.

Adoptive transfer homing experiments

WT and I-A/I-$E^{-/-}$ BM-DCs matured with LPS, and in some experiments $OVA_{323-339}$, overnight (as outlined in "Generation of Bone Marrow-Derived DCs"**)** were separately labeled with 5 µM CFSE (Sigma) or 10 µM CellTracker™ Orange CMRA (Invitrogen) in RPMI 1640 (Gibco) for 15 minutes at 37 °C. Cells were washed three times with PBS and incubated in DC medium for 30 - 40 minutes at 37 °C to quench unreacted dye. BM-DCs were subsequently washed with PBS and centrifuged over an FBS gradient to remove dead cells. Labeled WT and I-A/I-$E^{-/-}$ BM-DCs were mixed at a 1:1 ratio, resuspended in PBS and a total of 1.5 million BM-DCs were injected in two points into the ears of DTH-inflamed WT mice. After 40 - 44 hours, auricular LNs were harvested, stained with CD11c-APC, I-A/I-E-BV421, CD80-APC/Fire750 and CD86-BV605 and analyzed on a Beckman Coulter Cytoflex. After pre-gating on $CD11c^+$ cells, the ratio of $CFSE^+$ to $CMRA^+$ cells was determined. Dye combinations were changed for each experiment.

Statistical Analysis

All cell tracking data are presented as medians and all other results presented as mean plus SD. Data sets were analyzed using Prism 7 (GraphPad). Kruskal-Wallis test followed by post hoc analysis was used for multiple comparisons and Mann-Whitney U test for simple comparisons.

SUPPLEMENTAL REFERENCES

NITSCHKE, M., AEBISCHER, D., ABADIER, M., HAENER, S., LUCIC, M., VIGL, B., LUCHE, H., FEHLING, H. J., BIEHLMAIER, O., LYCK, R. & HALIN, C. 2012. Differential requirement for ROCK in dendritic cell migration within lymphatic capillaries in steady-state and inflammation. *Blood,* 120**,** 2249-58.

RUSSO, E., TEIJEIRA, A., VAAHTOMERI, K., WILLRODT, A. H., BLOCH, J. S., NITSCHKE, M., SANTAMBROGIO, L., KERJASCHKI, D., SIXT, M. & HALIN, C. 2016. Intralymphatic CCL21 Promotes Tissue Egress of Dendritic Cells through Afferent Lymphatic Vessels. *Cell Rep,* 14**,** 1723-34.

TEIJEIRA, A., HUNTER, M. C., RUSSO, E., PROULX, S. T., FREI, T., DEBES, G. F., COLES, M., MELERO, I., DETMAR, M., ROUZAUT, A. & HALIN, C. 2017. T Cell Migration from Inflamed Skin to Draining Lymph Nodes Requires Intralymphatic Crawling Supported by ICAM-1/LFA-1 Interactions. *Cell Rep,* 18**,** 857-865.

VIGL, B., AEBISCHER, D., NITSCHKE, M., IOLYEVA, M., ROTHLIN, T., ANTSIFEROVA, O. & HALIN, C. 2011. Tissue inflammation modulates gene expression of lymphatic endothelial cells and dendritic cell migration in a stimulus-dependent manner. *Blood,* 118**,** 205-15.

ZAL, T., VOLKMANN, A. & STOCKINGER, B. 1994. Mechanisms of tolerance induction in major histocompatibility complex class II-restricted T cells specific for a blood-borne self-antigen. *J Exp Med,* 180**,** 2089-99.
